# Aetiologies of basic biliteracy difficulties in Hong Kong Chinese children: A latent profile analysis with twin data

**DOI:** 10.1007/s11881-025-00355-w

**Published:** 2025-12-05

**Authors:** Shuting Huo, Anna Jia-Jun Zhang, Mo Zheng, Urs Maurer, Catherine McBride, Tomohiro Inoue

**Affiliations:** 1 Department of Psychology, The Chinese University of Hong Kong, Shatin, Hong Kong S.A.R China; 2 Department of Life Sciences, Beijing Normal-Hong Kong Baptist University, Zhuhai, China; 3 Department of Human Development and Family Science, Purdue University, West Lafayette, USA

**Keywords:** Aetiology, Biliteracy, Twin study, Latent profile analysis

## Abstract

The present study examined 1) basic biliteracy difficulty subtypes in Chinese (L1) and English (L2) Hong Kong Chinese children, 2) genetic-versus-environmental aetiologies for the subtypes, and 3) the moderating role of SES and school language use in the aetiologies. Four hundred fifty-six twin pairs (91.08 ± 13.20 months, 50% female) were assessed on Chinese and English word reading and spelling skills. Results of latent profile analysis identified four biliteracy profiles: 51.2% Chinese-dominant learners (CDL), 17.0% poor biliterate learners (PBL), 12.4% English-poor Chinese-dominant learners (EPCDL), and 19.4% English-dominant learners (EDL). Focusing on the difficulty profiles, results of familial resemblance showed that the additive genetic factor (55%) contributed more than the environmental factors to the probability of PBL, while the shared environmental factors (56%) contributed more than the genetic (25%) and unique environmental factors to the probability of EPCDL. Low SES was associated with a higher probability of PBL and EPCDL, and it exacerbated the genetic contribution to both difficulty profiles. The current findings suggest the influence of environmental factors underlying basic biliteracy difficulties, although we cannot rule out the possibility of high heritability, which appears to be the case for both L1 Chinese and L2 English.

**Supplementary Information:**

The online version contains supplementary material available at https://doi.org/10.1007/s11881-025-00355-w.

Learning to be biliterate has become a reality for many children globally. Basic biliteracy refers to the ability to read and spell words in two languages, such as alphabetic English and morphosyllabic Chinese (Zhang & Wang, 2023). Many children in biliterate societies are struggling with the task of mastering basic biliteracy skills. A debatable issue is whether the difficulties with word reading and spelling are linguistically universal or specific, that is, whether someone with difficulty in reading and spelling in one language will have difficulty in another (also referred to as the Linguistic Coding Difference Hypothesis; Sparks, 1995), or whether someone could have difficulty in one language but not in another (Miller-Guron & Lundberg, 2000; Oren & Breznitz, 2005). The latter perspective is particularly plausible in a context where one’s first and second languages feature contrastive orthographies, such as Chinese and English. Motivated by this rationale, the pioneering studies on word reading and spelling difficulties in the Chinese-English bilingual context have indicated both associations and dissociations of basic biliteracy difficulties, yielding different difficulty subtypes (e.g., Cheah et al., 2023; Tong et al., 2015).

However, there are some important questions that remain unanswered. First, how dissociative and associative biliteracy difficulty profiles are situated in the general biliterate population remains unclear. Thus, we still do not know whether previously identified distinct biliterate difficulty subtypes emerge as naturally occurring groups or are embedded in larger homogeneous group(s) of typical development. Importantly, from a methodological perspective, there is a consensus that selecting children with difficulties by imposing a set of arbitrary cutoff scores may lead to a loss of information (Erbeli et al., 2018; Fletcher, 1985; see also Hemelstrand & Inoue, 2025). Instead, it is recommended to include the general population when investigating reading (spelling) difficulties. To address these issues, the present study conducted latent profile analysis (LPA) to examine the heterogeneity of basic biliteracy in an unselected sample of Chinese-English bilingual twins in Hong Kong. Specifically, we sought to identify the different difficulty profiles suggested in previous literature (see below for a review).

Moreover, how these biliteracy difficulty profiles stem from genetic-versus-environmental origins is still largely unknown. It is advocated that the conceptualization of reading (spelling) difficulties needs to be evaluated by examining the corresponding aetiologies (Erbeli et al., 2018; Fletcher, 1985; Xie et al., 2022). We examined familial resemblance on profile memberships and posterior probabilities to reveal the genetic and environmental aetiologies underlying different basic biliteracy profiles. In addition, there are strong relations between low SES and poor word reading and spelling in both first and second languages (Liu et al., 2016; McBride-Chang et al., 2012). Language learning experiences, such as English as the medium of instruction in school, have also been shown to explain a substantial amount of variance in English reading and reading-related skills (Hu & Catts, 1998; Huo et al., 2022). In addition to exerting direct effects, these environmental factors may moderate the pathways by which genetic factors contribute to individual traits, a concept often referred to as the G × E (gene and environment) interaction (Plomin, 1990). In light of this, we examined whether SES and school language use moderate the genetic contributions to different basic biliterate profiles. The findings could potentially inform the definition of basic biliteracy difficulties in bilingual contexts.

## Basic biliteracy difficulties

Basic biliteracy difficulty is broadly defined as difficulties in word-level reading and spelling in two languages (Zhang & Wang, 2023), and dyslexia is the most common form of it. Dyslexia is defined as a decoding difficulty, specifically the difficulty of mastering the relationships between the spelling patterns of words and their pronunciations (Catts et al., 2024). It is characterized by difficulties with accurate and/or fluent word recognition and by poor spelling and decoding abilities. Given the similarity, literature on dyslexia can inform the current study about basic biliteracy difficulties.

Many studies on word reading/spelling difficulties in biliterate learners have revealed that there are different subtypes within the population in terms of weaknesses and strengths between one’s first and second languages (evidence for Chinese-English learners: Cheah et al., 2023; Li et al., 2018a, 2018b; Tong et al., 2015; evidence for biliterate learners of other languages: Miller-Guron & Lundberg, 2000; Van Der Leij & Morfidi, 2006). The *associative* subtype refers to those who have difficulty in both languages, whereas the *dissociative* subtype refers to those who have difficulty in one language but are intact in the other. In the case of Chinese-English biliterate children, Tong et al. (2015) adopted the 25th percentile as the cutoff criterion for difficulty; the results showed that 57% of Grade 5 children with Chinese reading difficulties did not suffer from difficulties in English reading; similarly, 50% of children with English reading difficulties had no problem reading Chinese. They also found that about 15% of Grade 2 and 5 children screened had reading difficulties in both languages. Li et al., (2018a, 2018b) screened a sample of Chinese children (*N* = 962) for English word reading difficulty with a different set of criteria (below 25th for difficulty and above 30th for typical performance). Using the criteria, they found that 32% of children with English difficulty did not have Chinese reading problems, and 43% had additional Chinese reading difficulty. Similarly, the evidence for associative and dissociative subtypes exists for spelling difficulty. Using the 25th percentile, the same as Tong et al., (2015), Cheah et al., (2023) identified 8–13% of Grade 2–3 children with only spelling problems in Chinese but not English, 9–10% with problems spelling English but not Chinese, and 11–16% with spelling difficulties in both languages.

Different biliteracy difficulty subtypes are shown to have distinct but partially overlapping profiles of cognitive-linguistic skills. For example, phonological skills have been shown to distinguish children with and without English literacy difficulties (e.g., Cheah et al., 2023; Li et al., 2018a, 2018b), whereas non-phonological skills, such as morphological awareness and delayed copying may be more closely associated with Chinese reading and spelling difficulties (e.g., Tong et al., 2015). Despite the documented differences at the cognitive level, neural abnormalities showed larger overlap across difficulty subtypes, which involve alterations of left-hemisphere areas such as the left supramarginal gyrus (Li et al., 2018a, 2018b), left precuneus, and left dorsal inferior frontal gyrus (Cao et al., 2021). The overlapping reading neural network suggests similar biological underpinnings across various basic biliteracy difficulty subtypes. Studies on dyslexia have partially supported this cross-linguistic universality account of word reading (spelling) difficulties, showing that the heritability of dyslexia is similar across languages, approximately 70% (Grigorenko, 2004). The author reviewed twin studies on developmental dyslexia across languages and showed that genetic factors contribute to anywhere between 53 to 91% of the variance in word reading and spelling.

In addition to the contrastive orthographies that may contribute to different biliteracy difficulty subtypes, Lundberg (2002) advocated for a comprehensive model that considers social, affective, and environmental factors, particularly for second language basic literacy. He further posits that these factors might explain most of the individual variation, which could mask the influence of linguistic differences between L1 and L2 in children with basic biliteracy difficulties. In the context of English and Chinese, several studies showed that environmental disadvantages such as lower SES and little English use at school are linked to particularly poor performance in L2 English. In a study with a sample of typically developing children, Liu et al. (2016) showed that SES significantly contributed to L2 English reading after cognitive-linguistic skills were controlled for, but this unique effect was not observed for L1 Chinese reading. In struggling readers, McBride-Chang et al. (2012) found that poor Chinese readers with additional English reading difficulties were children of mothers with lower education levels, whereas those without English difficulties did not show such results.

## The environmental and genetic influences of English and Chinese literacy skills

To date, two studies with Chinese-English bilingual twin samples have examined the environmental and genetic contributions to the language-universal and English-specific aspects of biliteracy-related skills (Wong et al., 2014; Xie et al., 2022). Wong et al. (2014) showed that genetic contribution explained the majority of variance in both L1 Chinese word reading (76%) and L2 English word reading (53%). Furthermore, both studies indicated significant genetic overlaps between parallel L1 Chinese and L2 English skills, including word reading, phonological processing, morphological awareness, speech discrimination, and vocabulary. The bivariate heritability, the amount of phenotypic covariance between English and Chinese explained by genetic factors, ranged from 37 to 85%. In addition to the shared genetic influence, Wong et al. (2014) showed a significant English-specific genetic contribution, not overlapping with the genetic contribution shared with Chinese, that explained 11–49% of the variance in English word reading.

Regarding environmental influences, Wong et al. (2014) showed overlapping individual environmental influences for word reading and speech discrimination. They also found English-specific individual environmental contributions to all the English reading and linguistic skills in the study, ranging from 26–89%. These findings attested to the cross-linguistic universality of literacy and language acquisition by showing the aetiological overlap. In addition, the findings also provide evidence of language specificity by showing specific genetic and environmental contributions to L2 English reading and related skills, of which the environmental contributions are particularly pronounced.

Among the salient environmental influences, SES and school language use are identified as environmental factors that partially moderate the common environmental contribution to various English and Chinese language skills in a Hong Kong bilingual twin sample (Xie et al., 2022). Indeed, in addition to their direct effects on basic biliteracy outcomes, these environmental factors may alter the extent to which genetic factors contribute (i.e., G × E interaction). The vulnerability-stress model (Rende & Plomin, 1992) of developmental disorders (DD) posits that a less supportive familial environment may lead to greater genetic liability from detrimental DD-susceptibility genes’ alleles, which would remain undetected in more supportive environments. This model has been empirically supported by some studies of children with dyslexia in their first language; lower SES was linked to an increased, albeit weak, effect of a risk gene (DYX1C1-1259C/G; Mascheretti et al., 2013). However, the vulnerability-stress model has not been examined for basic biliteracy difficulties, so it remains unanswered whether environmental disadvantage would lead to greater genetic contributions to basic biliteracy difficulties and whether this interaction varies across difficulty subtypes.

## The current study

The aim of this study was threefold. First, we sought to identify different basic biliteracy difficulty subtypes in a general biliterate population of Chinese-English bilinguals in Hong Kong. Previous studies used a variable-centred approach to investigate the aetiologies of biliteracy and literacy-related skills. Although their findings provide important insights into the similarities and distinctiveness between L1 Chinese and L2 English basic biliteracy skills (Wong et al., 2014; Xie et al., 2022), they do not directly inform the aetiologies of basic biliteracy difficulties or language dominance in a biliterate setting. We used latent profile analysis (LPA) to classify the general population in search of those who have basic literacy difficulties in one language or both. There are several advantages to using LPA to identify biliteracy difficulties. First, no specific cutoff scores are used to identify groups (i.e., latent profiles). Rather, all test scores are used simultaneously to determine the probability that an individual is in each latent profile in the model. Second, LPA allows us to account for the heterogeneity in performance levels across different aspects of literacy skills among an unselected sample of children. Therefore, the findings can situate the linguistically specific difficulty subtypes in relation to the general biliterate population regarding the relative strengths and weaknesses between L1 Chinese and L2 English. The theory and evidence reviewed above support both dissociation and association between reading and spelling difficulties in L1 Chinese and L2 English (e.g., Tong et al., 2015). Therefore, we expected to find associative biliteracy profiles where the performances are similar between Chinese and English, and dissociative profiles where the Chinese and English literacy abilities are contrastive. However, we did not formulate hypotheses for the specific number of profiles in the general biliterate population. Thus, we adopted the exploratory approach of LPA.

The second aim was to examine the extent to which different biliteracy profiles are influenced by genetic and environmental factors. We expected significant genetic and environmental contributions to different basic biliteracy profiles. In addition, we hypothesized different aetiologies for different basic biliteracy profiles. Specifically, the profiles with English difficulties would have more environmental contributions than those without. The third aim of the study was to examine the interaction between two environmental factors (SES and school language use) and genetic contributions. Based on the vulnerability-stress model (Rende & Plomin, 1992), we expected that genetic contributions to the probability of basic biliteracy difficulties would increase in a more disadvantaged environment (i.e., lower SES and less use of English in school).

## Method

### Participant

Participants were drawn from an ongoing longitudinal twin project in Hong Kong (Xie et al., 2022). Twins were considered eligible for the initial project if they were enrolled in a local kindergarten or elementary school. A total of 456 pairs of Hong Kong Chinese twins from Grade 1 to Grade 4 (*M*_age_ = 90.96 months, *SD* = 13.59) and their families participated in the study. A full description of sample characteristics is shown in the supplementary materials (Table S1). The sample was an unselected sample representing the general population of twin children in Hong Kong, covering the entire distribution of reading skills. Despite varying home language ratios, all families spoke Chinese as the dominant language, according to parents’ reports of home language use. All children went to publicly-funded or subsidized schools where the curricula followed the guidelines published by the Hong Kong Education Bureau. The medium of instruction was a mixture of Chinese and English; the ratio between Chinese and English varied across schools. Of the 456 pairs of twins, 195 were monozygotic (MZ; 105 female pairs), 259 were dizygotic (DZ; 134 mixed-sex pairs; 54 female pairs), and two were of an undetermined zygosity and were excluded from the analysis.

### Measures

Our analyses used the data on several measures for different purposes. Specifically, word reading and dictation tasks in Chinese and English were used as classification measures in latent profile analysis (LPA); Chinese word reading fluency and English vocabulary knowledge were used as external validation measures to examine the validity of the profiles identified in LPA; family socioeconomic status (SES) and school language use were used as potential moderators to examine the gene-environment (G × E) interaction. Each of these measures is described below.

### Classification measures

#### Chinese word reading

The participants were given items adapted from the Hong Kong Test of Specific Learning Difficulties in Reading and Writing (Ho et al., 2007), which included 150 two-character words. The items were arranged in order of increasing difficulty. The task was stopped when the child failed to read 15 consecutive items. One point was given for the correct recognition of both characters. The Cronbach’s alpha of this test in our sample was .95.

#### English word reading

The English word reading test used in the present study was developed by Tong and McBride-Chang (2010). There were 50 items in total. All words were selected from English textbooks used in Hong Kong kindergartens and primary schools. The test items were arranged in five levels in order of increasing difficulty. One point was given for each correct answer. The task was stopped when the child answered four consecutive items incorrectly at one level. The Cronbach’s alpha of this test in our sample was .96.

#### Chinese dictation

The children were asked to write down 20 two-character Chinese words. The words were arranged by character complexity as well as age of acquisition (Tong et al., 2009). A two-point scale was developed to code the children’s writing. One point was given if a child could write down at least one radical of the character, or s/he only made a minor error in one of the radicals (e.g., missing stroke, additional component), and two points were given if s/he could write the whole character correctly. Therefore, the maximum score for the task was 40. The Cronbach’s alpha of this test in our sample was .92.

#### English dictation

This task required the participants to write down 12 English compound words (e.g., < nighttime > , < seasick >). The stimuli were selected from English textbooks for primary school children in Hong Kong (Lo et al., 2018). The English words were presented orally to children. The scores were provided based on how many morphemes, or individual words, they wrote down correctly. A two-point scale was developed to code each item. One point was given for each correctly spelled morpheme or word within the compound word. For example, for the word < nighttime > , if the participant answered < nighttie > , the total score for this item was 1 (night is correct) + 0 (tie should be time). The maximum possible score for this task was 24. The Cronbach’s alpha for this task in our sample was .89.

### Validation measures

#### Chinese word reading fluency

This task was adapted from the 1-Minute Word Reading subtest of the Hong Kong Test of Specific Learning Difficulties in Reading and Writing for Primary School Students – Second Edition (HKT-P[II]; Ho et al., 2007). Children were asked to read 90 high-frequency two-character words as quickly and accurately as possible within one minute. The total number of words read correctly within one minute was recorded. If a child finished the task in less than one minute, the time duration to complete the task was also recorded. The score was calculated as (number of words read correctly * 60) / (duration in seconds). The maximum possible score was 90 or more, depending on the time to complete the task. The test–retest reliability of this test was .92.

#### English vocabulary knowledge

This test consisted of three subtests, including receptive vocabulary, expressive vocabulary, and word definition (McBride-Chang et al., 2008). Children were asked to name the pictures in the expressive task and select the picture that matched the word heard in the receptive task. Additionally, children were asked to provide a definition of a word orally presented to them. The receptive and expressive vocabulary items were selected from the Peabody Picture Vocabulary Test (PPVT-III; Dunn & Dunn, 1997). The total number of items in the test was 70. The Cronbach’s alpha of this test in our sample was .81.

### Moderator measures

#### Socioeconomic status (SES)

A composite score of SES was created using principal component analysis, which is based on the primary component that explained the most variance in a dataset of mother education level, father education level (7-point scales; 1 = *Secondary 3 or below*, 2 = *Secondary 4 to 5*, 3 = *Secondary 6 to 7*, 4 = *Associate degree*, 5 = *Bachelor’s degree*, 6 = *Master’s degree,* and 7 = *Doctorate degree*), and family income (6-point scales from 1 = *HK$10,000 or below*, 2 = *HK$10,001–20,000*, 3 = *HK$20,001–30,000*, 4 = *HK$30,001–40,000*, 5 = *HK$40,001–50,000*, and 6 = *HK$50,001 or above*). Standardized scores were used in the analysis.

#### School language use

Following our previous practice (Xie et al., 2022), the ratio of English and Chinese language use at school was calculated based on parents’ responses on the number of subjects taught in English versus Chinese. For example, the ratio is 0.5 if a parent reported that two subjects were taught in English and four subjects were taught in Chinese (Mandarin or Cantonese). Twin pairs had the same school language use with each other.

### Procedure

A saliva sample with a DNA kit was collected from each participant for zygosity testing to determine whether the twins were monozygotic or dizygotic. The language tests were administered at participants’ homes by trained experimenters, and the instructions were all in Chinese.

### Statistical analysis

#### Classification of biliteracy profiles

First, latent profile analysis (LPA) was performed to identify the latent profiles and their membership in terms of Chinese and English biliteracy in Chinese primary school children. The current sample included children of a wide age range, from 7 to 11 years old, which could influence the classification results in two ways: 1) age could be a covariate of the classification variables, and 2) age could be a predictor of class membership. Therefore, age was treated as a covariate in the three-step approach recommended by Asparouhov and Muthén (2014). Specifically, to address the first possibility, raw scores on the four classification measures were regressed on age, age second power (age^2^), and months of schooling. The standardized residuals were then used to estimate the best-fitting unconditional model (Wong et al., 2014). To address the second possibility, the posterior probability of each latent class from the best model identified in the previous step was calculated and regressed on age and age^2^. In the final step, the profile membership was estimated based on the age-adjusted posterior probability.

Although we hypothesized that we would find associative and dissociative biliteracy difficulty profiles, the heterogeneity in the rest of the population was unknown. Therefore, we used an exploratory approach to determine the optimal number of profiles (Muthén & Muthén, 2008–2017). Regarding the variance/covariance structure, we followed Peugh and Fan’s (2013) recommendation to allow for the dependence of profile indicators within profiles and variance heterogeneity across classes. They mentioned that the LPA assumptions of local independence of profile indicators and variance homogeneity could lead to model misspecification. This point was further supported in that the fully variant model performed better than the partially-variant and non-variant models, as shown in the supplementary materials (see Table S3). We fitted seven fully variant models to the data, increasing the number of profiles defined from one to seven profiles. Following previous practice with twin samples (Hart et al., 2016), the model with the clustering structure of family was estimated to account for the twin similarity (Muthén & Muthén, 2008–2017).

Model fit was assessed using the Bayesian information criterion (BIC), Sample-Size Adjusted BIC (SABIC), and the bootstrap likelihood ratio test (BLRT). Because relying on only empirical fit indices for model selection can lead to overinterpretation of the empirical results (Lubke & Muthén, 2005), we used theory and previous findings to guide our selection of the optimal profile solution. Specifically, we analyzed the transition matrices, which show how many participants from a given class of a *k*-class model are assigned to each of the profiles in a *k* + 1-class model and, therefore, reveal the stability of profiles across solutions with increasing numbers of profiles. The transition matrix can inform how the profiles are formed. A more complex solution should be adopted if it provides more information about the heterogeneity in the population aligned with theory (Lubke & Muthén, 2005). In addition, we followed the recommendation by Jung and Wickrama (2008) and considered the entropy value (greater than .8, or closest to 1.0; see also Ramaswamy et al., 1993), the percentage of individuals in each class (no less than 1% of the total participant count in a given class), and posterior probabilities (close to 1.0). High posterior probabilities indicate that there is high confidence that an individual assigned to a given class belongs to that class.

The LPA was performed using Mplus (Muthén & Muthén, 1998–2017). No missing data was identified on the four classification measures. After transforming the raw scores to *z*-scores, two cases of Chinese dictation scores were more than three standard deviations below the mean, and two cases of Chinese word reading were more than three standard deviations above the mean. These scores were recoded to be missing, given the potential for extreme scores to skew the means of the latent profiles, as well as possibly distorting the *p*-value of the BLRT (Nylund et al., 2007).

We validated the profiles identified in the LPA by comparing them on the external validation measures (i.e., Chinese word reading fluency and English vocabulary knowledge). Multivariate analysis of variance (MANOVA) was conducted in the *restatis* package in R (R Core Team, 2023), with the validation measures as the dependent variables and class membership as the independent variable. Post hoc comparisons were performed using the Bonferroni-corrected independent sample *t*-test.

#### Influence of genetic and environmental factors

**Fig. 1:**
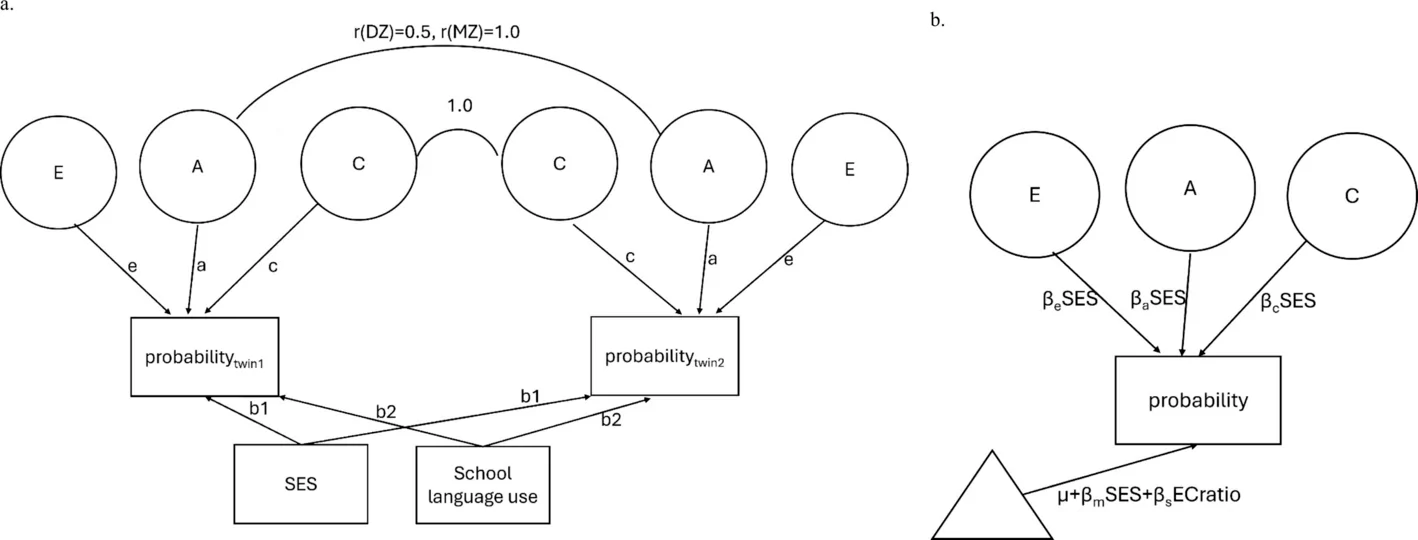
Theoretical behavioural-genetic model with covariates (**a**) and moderated behavioural-genetic model (**b**)

Next, we computed the correlations for class membership probabilities for the MZ and DZ pairs, respectively, and used these to obtain the heritability of class membership. We employed a univariate model adapted from Petrill et al. (2004) (see Fig. 1a) to examine the approximate proportion of the phenotypes (age-adjusted probability of basic biliteracy profiles) explained by identified shared environment (F) factors along with additive genetic effects (A), shared environmental effects (C), and nonshared environmental effects (E). SES and school language use were the F factors in this study. The total proportion explained by shared environment was the sum of the C and F factors. If the confidence intervals of profile estimates did not overlap, this indicated a statistically significant difference between the profile estimates. These analyses were performed using the *OpenMx* package (Boker et al., 2011) in R. The ACE analysis of class probabilities was further complemented by the analysis of class membership, which included the affected-status agreement of profile membership (Hart et al., 2016) and MZ/DZ odds ratio of twin concordance (Neuman et al., 2001). The results are presented in the supplementary materials (see Table S2), which supported the findings of our ACE analysis regarding familial resemblance.

#### Gene-environment (G × E) interactions

We employed the G × E model (Purcell, 2002), where the A, C, and E main effects were accompanied by the potential moderator measures (i.e., SES and school language use; see Fig. 1b). The G × E models were built separately for SES and the English/Chinese ratio (EC ratio) of school language use as moderators. Before running the models, missing values were imputed for SES (*n* = 149, 16%) and school language use (*n* = 309, 34%) using the maximum-likelihood method (Van Buuren, 2018). For cases where the imputed values differed from those of the twin counterparts, the average of the two values was used for further analysis. Unstandardized results are reported below because standardized solutions have been found to lose information about the source of variance (Purcell, 2002). Statistical significance of a moderating effect was obtained by comparing the fully moderated model (all three factors are moderated) to a model without the moderating effect.

## Results

### Basic biliteracy profiles

The descriptive statistics of all the classification and validation measures and the correlation coefficients between them are shown in Table 1. The fully variant model of seven profiles did not converge. Therefore, the model fit indices, entropy, and posterior probabilities for the one- to six-profile solutions are reported in Table 2. The results of BLRT showed that there was no significant difference in the model fit between the four- and five-profile solutions, suggesting that the five-profile solution fitted the data no better than the four-profile solution. In addition, the values of SABIC and BLRT suggested that the four-profile solution showed the best fit. Since the current analysis approach was exploratory, the three possible solutions (i.e., three-, four-, and five-profile solutions) were considered as shown in the supplementary materials (see Table S4).

**Table 1:** Descriptive statistics and correlations for the classification and external measures

	1. Age	2. CWR	3. EWR	4. CDICT	5. EDICT	6. COM	7. EVOC	8. SES	9. ECratio
	Correlation coefficients								
1		.39	.68	.57	.43	.54	.22	-.13	.09
2	.06		.48	.76	.48	.83	.22	-.07	.00
3	.00	.31		.47	.92	.47	.80	.39	.34
4	.12	.61	.31		.49	.66	.26	.01	.01
5	.00	.27	.90	.32		.46	.76	.39	.37
6	.11	.59	.25	.38	.23		.24	.00	-.01
7	.00	.12	.80	.17	.77	.18		.50	.35
8	-.13	.03	.48	.09	.49	.08	.54		.16
9	.09	-.07	.34	-.04	.37	-.05	.33	.16	
	Means and standard deviations								
Mean_raw_	91.01	70.45	19.40	25.05	8.15	53.31	24.10	0.21	0.36
*SD*_raw_	13.62	35.58	15.59	8.93	7.34	12.01	21.35	1.46	0.49
*n*	904	904	904	904	904	898	898	755	505

The correlation coefficients in the upper panel are yielded from raw scores, and the lower panel from standardized scores. *CWR* Chinese word reading, *EWR* English word reading, *CDICT* Chinese dictation, *EDICT* English dictation, *COM* Chinese one minute word reading, *EVOC* English vocabulary knowledge, *raw* raw score, *ECratio* English/Chinese ratio of school language use

**Table 2:** Model fit indices and classification accuracy

Profiles	AIC	BIC	SABIC	BLRT_val	BLRT_p	Entropy	Prob_min	Prob_max
1	8217.29	8284.59	8240.13	NA	NA	1.00	1.00	1.00
2	7834.40	7973.79	7881.70	412.90	0.00	0.87	0.91	0.97
3	7706.10	7917.60	7777.87	158.29	0.00	0.80	0.91	0.92
4	7653.84	7937.44	7750.07	82.26	0.00	0.76	0.67	0.94
5	7651.88	8007.58	7772.57	44.03	0.43	0.78	0.72	0.90
6	7625.86	8053.67	7771.02	82.39	0.00	0.72	0.73	0.89

*AIC* Akaike Information Criterion, *BIC* Bayesian Information Criterion, *SABIC* Sample Adjusted BIC, *BLRT* Bootstrap Likelihood Ratio Test

The four-class solution was selected for the unconditional model based on the model fit, although the classification accuracy indicated by the entropy value (0.76) was slightly lower than the conventional threshold (0.8). Transition matrices, presented in the supplementary materials (see Table S5), showed that the profiles in the four-profile solution were stable, with 83.8% of cases staying in the same membership across solutions on average. The corresponding stabilities were 84.2% for the five-class solution and 76.1% for the three-profile solution. In addition, the four-profile solution revealed more about the heterogeneity of the population, aligning with the dissociation hypothesis of English and Chinese basic biliteracy difficulty, as both associative and dissociative basic biliteracy difficulty profiles were identified.

**Fig. 2:**
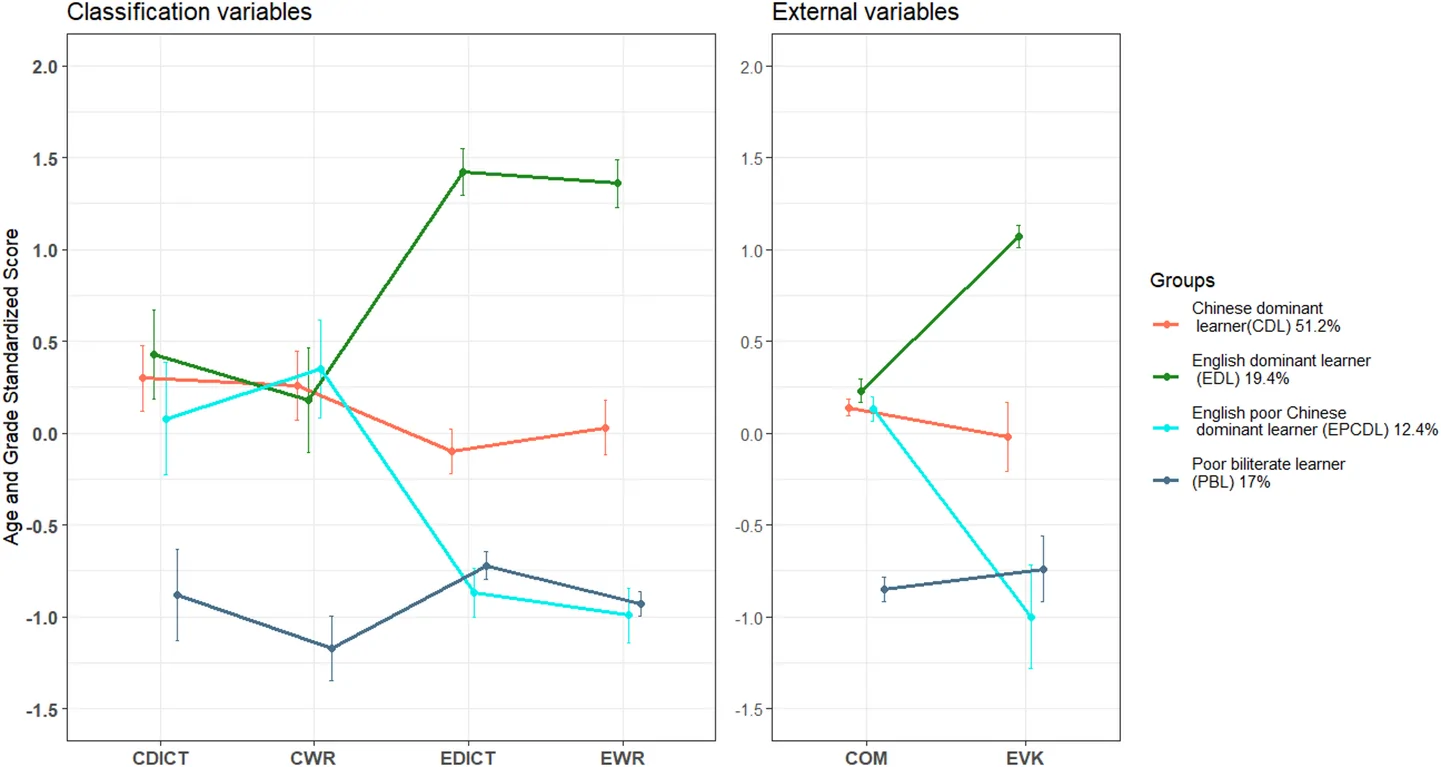
Profiles of the final age-adjusted four-class solution from the latent profile analysis. Note. CWR, Chinese word reading; CDICT, Chinese dictation; EWR, English word reading; EDICT, English dictation; COM, Chinese one-minute reading; EVK, English Vocabulary Knowledge. The thin bars represent 95% confidence intervals

The final solution adjusted for age is presented in Fig. 2. For the final four-profile solution, 17.8% of the sample was assigned to profile 1, and they showed low performance (0.5 *SD* below the grade- and age-adjusted average) in all four measures. Thus, profile 1 was labeled as “poor biliteracy learners” (PBL). 41.7% of the children were assigned to profile 2, which was the normative group and had slightly lower performance in English than in Chinese. Thus, profile 2 was labeled as “Chinese-dominant learners” (CDL). 21.9% of the children were assigned to profile 3, and in contrast to profile 2, they showed higher performance in English than in Chinese literacy skills. Moreover, their Chinese performance was above the average level. Thus, profile 3 was labeled as “English-dominant learners” (EDL). Finally, 18.6% of children were assigned to profile 4, who scored substantially below average in the English measures but above average on the Chinese measures and thus were labeled as “English-poor Chinese-dominant learners” (EPCDL).

The four-profile solution also showed external validity. The four profiles differed significantly from each other on other literacy-related variables, namely Chinese word reading fluency and English vocabulary knowledge. For Chinese word reading fluency, the performance of PBLs was significantly lower than the other three groups (all *p* < .05), and there was no significant difference among the other three groups. For English vocabulary knowledge, EDLs performed significantly better than the other three groups; CDLs performed significantly better than PBLs and EPCDLs; PBLs outperformed the EPCDLs (all *p* < .05). The performances of these groups are shown in the supplementary materials (see Table S6).

### Influence of genetic and environmental factors

To delineate the genetic versus environmental contributions to individual differences in the probability of being in each group, the ACE analysis results, along with their corresponding univariate estimates and 95% confidence intervals (CI), are presented in Table 3. The overall goodness-of-fit of the model was deemed satisfactory for each variable of probability, with *p*-values exceeding .05. For the PBL group, we found the pivotal role of additive genetic effects (A) in accounting for a substantial proportion of variance 55%, while the contributions from shared (C) and nonshared (E) environmental factors were less pronounced, 28% and 17%, respectively. Among the shared environmental influences, SES and school language use explained 3% of the variance, whereas the contribution of school language use was not significant. Within the PBL group, the additive genetic influence (A) was significantly greater than the non-shared environmental influence (E).

**Table 3:** Results of familial resemblance analyses and ACE model with covariates

Profile	Between-pair correlation	A	C	E	SES	School language use
Poor biliterate learners (PBL)	MZ: 0.794 DZ: 0.549	0.55 [.38;.74]	0.24 [.07;.40]	0.17 [.09;.23]	0.03 [.01; .05]	0.01 [.00;.10]
Chinese-dominant learners (CDL)	MZ: 0.627 DZ: 0.479	0.37 [.13;.60]	0.26 [.04;.45]	0.14 [.00;.31]	0.00 [.00; .01]	0.24 [.09;.42]
English-dominant learners (EDL)	MZ: 0.803 DZ: 0.761	0.18 [.01;.35]	0.55 [.41;.68]	0.00 [.00;.03]	0.08 [.05; .11]	0.19 [.12; .26]
English-poor Chinese-dominant learners (EPCDL)	MZ: 0.704 DZ: 0.502	0.25 [.03;.50]	0.36 [.15;.55]	0.19 [.00;.32]	0.05 [.03; .08]	0.15 [.04; .33]

Shared environmental influence is the sum of c factor, *SES* and school language use. *A* variance explained by additive genetic factors, *C* variance explained by common environmental genetic factors, *E* variance explained by unique environmental genetic factors. *MZ* Monozygotic twins, *DZ* Dizygotic twins

For the EDL group, the shared environmental effect (C) emerged as the dominant contributor, explaining the majority of variance (82%) compared to the additive genetic (18%) influences. SES and school language use both made significant contributions, explaining 8% and 19% of the total variance, respectively. Similar to the EDL group, the EPCDL group showed an impact of shared environmental factors (56%) on L2 English literacy skills, among which SES and school language use explained 5% and 15% of the total variance, respectively. 25% of the variance in EPCDL probability was explained by the additive genetic effect, leaving the remaining 19% explained by non-shared environment. Finally, as for the CDL group, our results showed a relatively balanced contribution of the additive genetic (37%), shared environmental (50%), and nonshared environmental (13%) factors. School language use significantly explained 24% of the total variance, whereas the contribution of SES was not significant.

### Gene-environment (G × E) interactions

**Fig. 3:**
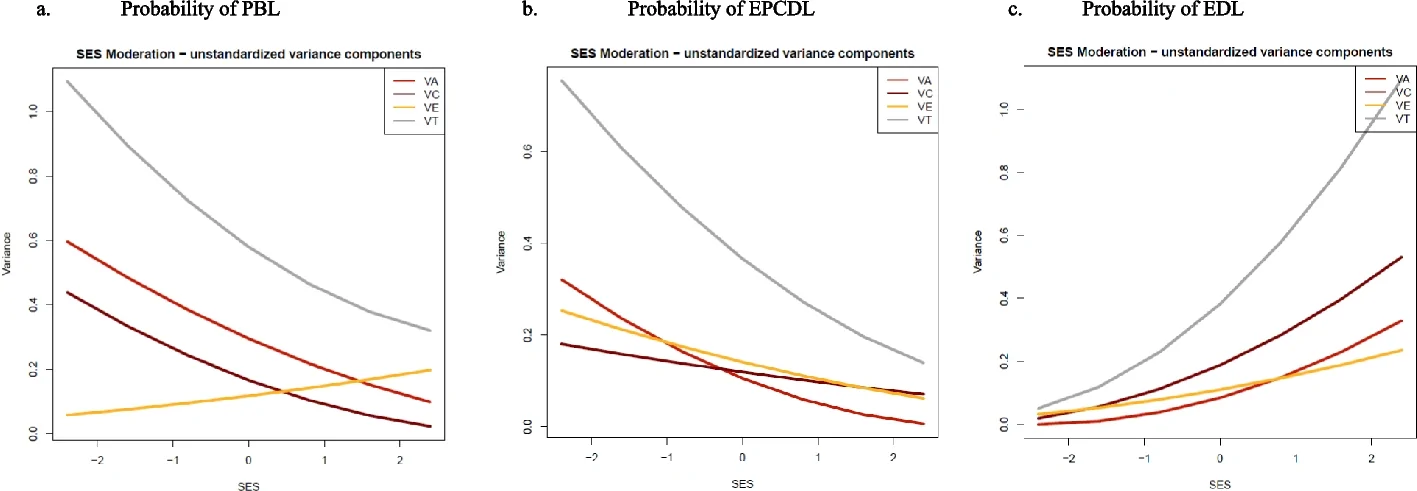
ACE contributions by SES for **a**) poor biliterate learners (PBL), **b**) English-poor Chinese-dominant learners (EPCDL), and **c**) English-dominant learners (EDL). Note. VA, variance explained by additive genetic factors; VC, variance explained by common environmental genetic factors; VE, variance explained by unique environmental genetic factors; VT, total variance explained

The results of the moderated ACE model are shown in Table 4 and Fig. 3. None of the moderated models fit the data better than the model without a moderator for the CDL group, suggesting no moderating effect of SES and school language use. In addition, all of the moderated models using school language use as a moderator failed to converge with acceptable accuracy. Therefore, we focus here on the moderating effect of SES for the PBL, EPCDL, and EDL groups. The fully moderated model for the PBL group showed that higher SES was significantly linked to reduced genetic and shared environmental effects, but to increased non-shared environmental effects (see Fig. 3a for a visual illustration of the interaction). The χ^2^ difference test showed that the fully moderated model fit the data better than the models with fewer moderating effects (Δχ^2^ = 6.56–17.18, Δ*df* = 1, all *p* < .05). The model for the EPCDL group showed that higher SES was significantly linked to reduced genetic and non-shared environmental effects (see Fig. 3b). However, the χ^2^ difference test showed that the fully moderated model fit the data no better than the model without moderating effects on a shared environmental effect. Finally, the model for the EDL group showed that higher SES was significantly linked to increased contribution of all three effects (see Fig. 3c). The χ^2^ difference test showed the fully moderated model best fit the data (Δχ^2^ = 6.51–30.80, Δ*df* = 1, all *p* < .05). Overall, these results suggested that genetic contributions to the probability of PBL and EPCDL decreased with an increase in SES, but the pattern is reversed for EDL, i.e. the genetic contribution increased with SES.

**Table 4:** Estimated parameters in the ACE model with SES as moderators

	Poor biliterate learners		English-poor learners		English-dominant learners	
Parameters	Estimate	*SE*	Estimate	*SE*	Estimate	*SE*
β_μ_SES	−0.12	0.02	−0.13	0.02	0.14	0.02
β_μ_EC	−0.21	0.05	−0.08	0.06	0.67	0.07
μ	−0.04	0.03	−0.08	0.04	−0.45	0.04
a	0.32	0.07	0.54	0.05	0.29	0.06
c	0.35	0.06	−0.41	0.07	0.43	0.04
e	0.38	0.02	0.34	0.02	0.33	0.02
βaSES	−0.10	0.03	−0.10	0.03	0.12	0.03
βcSES	−0.03	0.03	0.11	0.04	0.12	0.02
βeSES	−0.05	0.01	0.04	0.01	0.06	0.01

*μ* the mean of profile probability, *a* the path of additive genetic effect, *c* the path of common environmental effect, *e* the path of individual environmental effect, β_μ_SES the path of SES on μ, *βaSES* the path of SES on a, *βcSES* the path of SES on c, *βeSES* the path of SES on e, β_μ_EC the path of English Chinese ration in school on μ

## Discussion

In this study, we examined basic biliteracy difficulty subtypes in a sample of L1 Chinese and L2 English Hong Kong Chinese twin children, as well as genetic-versus-environmental aetiologies for the subtypes. We also examined the moderating role of SES and school language use in the aetiologies. Previous studies with Chinese-English bilingual children have demonstrated the subtypes of basic biliteracy difficulties, characterized by both associative and dissociative difficulty profiles (e.g., Cheah et al., 2023; Tong et al., 2015). The present study extends previous work by revealing 1) the heterogeneity of Chinese-English basic biliteracy in an unselected sample of a biliterate population, 2) the different environmental-versus-genetic aetiologies corresponding to different biliteracy profiles, and 3) the interaction between SES and genetic contributions to profile probabilities. We identified two biliteracy difficulty profiles, as well as two profiles with different dominant languages among typically achieving children, supporting the heterogeneity in the biliterate population in terms of strengths and weaknesses between one’s first and second languages. Focusing on the difficulty profiles, we showed that poor biliteracy learners (PBL) had the largest genetic contribution, while English-poor Chinese-dominant learners (EPCDL) had larger environmental contributions. Moreover, SES contributed to shared environmental influences for both PBL and EPCDL, whereas school language use contributed to them only for EPCDL. Finally, we found significant G × E interactions, indicating that lower SES was associated with greater genetic contributions to the probability of both PBL and EPCDL.

### Basic biliteracy profiles

Our results showed that Chinese-English biliterate children in Hong Kong are a heterogeneous population with multiple profiles in terms of relative strengths and weaknesses between Chinese and English basic (word-level) literacy skills. Two profiles with difficulties were identified, PBL and EPCDL, taking up 18% and 19% of the unselected sample, respectively. Although the raw percentage was higher than previous findings from studies using a score cutoff criterion (e.g., Cheah et al., 2023; Tong et al., 2015), the ratio of the two subtypes, which was approximately 1:1, was similar to previous findings. Taken together, these results suggest that the prevalence rates of associative-difficulty and second-language-difficulty-only subtypes are similar in a biliterate context.

In addition to the difficulty profiles, we found two profiles of typically developing biliterate children differing in language dominance. The profile of the largest size was the Chinese-dominant learners (CDL), who had Chinese word reading and spelling skills above the average level, but their English word reading and spelling skills slightly fell behind the grade average. The other profile of smaller size was the English-dominant learners (EDL). In contrast to CDL, EDL had better English literacy skills than their Chinese skills, and their performance in both languages was higher than the grade average, making them balanced biliteracy learners. These profiles of different language dominance echo previous studies classifying bilingual children on the dimensions of language use and proficiency (Bialystok et al., 2005; Hoff et al., 2021). Our results suggest that, similar to bilingualism, biliteracy is unlikely to be a dichotomous phenomenon (one is either biliterate or not). Instead, the construct of biliteracy is more likely to be continuous and multidimensional. It should be noted, however, that the current study focused only on performance in basic, word-level literacy skills. How literacy skills at a higher level (e.g., reading comprehension and writing composition) contribute to the construct of biliteracy remains unknown. Additionally, the literacy experience with the two languages may also be considered in the concept of biliteracy. The frequency and intensity with which biliterate children interact with a script may differ between their first and second languages, or between the orthographies they learn. Biliteracy experience is an understudied area and should receive more attention in future studies.

In this study, we sought to examine whether the previously identified biliteracy difficulty profiles emerge as distinct groups or are embedded in larger homogeneous groups. The results of our latent profile analysis (LPA) suggested that associative difficulty and English-L2-poor-only subtypes were naturally occurring groups, whereas the Chinese-L1-poor-only subtype may exhibit literacy skill patterns similar to, and thus be difficult to separate from, the English-dominant learners. To verify this interpretation, we performed an additional analysis using the previously adopted cutoff criteria of 25% (or *z* < −0.675; see e.g., Cheah et al., 2023) to identify the associative and dissociative difficulty profiles in the current sample. The difficulty membership yielded from the cut-off approach was cross-tabulated with the membership yielded from our final LPA model (see the supplementary materials, Table S7). The results showed that the majority (70%) of the English reading difficulty only group overlapped with the EPCDL group identified in our LPA, 63% of the Chinese reading poor only group overlapped with the EDL group, and 73% of the associative difficulty group overlapped with the PBL group. When we repeated the same additional analysis using the dictation task, the proportions of overlap were 51%, 51%, and 52% for the EPCDL, EDL, and PBL groups, respectively. Taken together, these results confirm our interpretation that the Chinese-poor-only subtype is largely embedded in and represents the lower extreme of the English-dominant learners.

### Influence of genetic and environmental factors

As we discussed above, our LPA showed that biliterate children are a heterogeneous population in terms of relative strengths and weaknesses between basic biliteracy skills in their first and second languages. Our twin sample further allowed us to investigate the underlying genetic versus environmental influences on the identified profiles. Significant aetiological differences were identified between the two biliteracy difficulty subtypes. Specifically, the heritability for PBL was 55%, which is similar to the heritability of dyslexia; previous twin studies in alphabetic languages have suggested that dyslexia is moderately heritable with an estimated heritability (h2) ranging from 40 to 60% (Fisher & DeFries, 2002). A similar range of heritability has been reported for word reading and spelling among general populations (h^2^ ~ 40–70%; e.g., Grigorenko, (2004)). The genetic contribution of PBL is also consistent with the significant bivariate heritability (53%) shared between Chinese and English word reading (Wong et al., 2014). The similarity across contexts speaks to the cross-linguistic universality of acquiring basic biliteracy. Across scripts and language status (monolingual or bilingual), genetic factors appear to be the primary cause of extremely and broadly low basic biliteracy achievement in modern societies where formal literacy instruction is abundant.

Despite the dominant role of genetic influences, a large proportion of individual variance for PBL was attributed to shared environmental factors, suggesting that factors at the family- or school-level also explain variance in one’s probability of having basic biliteracy difficulties in both languages. One identified environmental factor was SES; lower SES was found to be associated with a higher probability of PBL, whereas no such association was found for school language use. These results are consistent with previous evidence showing that children’s word reading difficulties in both L1 Chinese and L2 English were associated with lower maternal education levels (McBride-Chang et al., 2012). This suggests that lower SES may be particularly linked to the overall difficulty in acquiring basic biliteracy skills in both first and second languages. More importantly, lower SES was also associated with a higher contribution of genetic factors to PBL, as revealed in the significant G × E interaction (see Fig. 3). These findings are consistent with the findings of previous studies on dyslexia (Catts & Petscher, 2021; Mascheretti et al., 2013) and extend the vulnerability-stress model to children’s literacy development in a biliterate context. In particular, the genetic liability for basic biliteracy difficulties is more likely to manifest in a stressful environment (i.e., lower SES), whereas it is less likely to be detected in a protective environment (i.e., higher SES; see Catts & Petscher, 2021, for a relevant discussion for dyslexia).

In contrast to the aetiology of PBL, English-poor Chinese-dominant learners (EPCDL) stemmed from a different composition of genetic and environmental factors, particularly downplaying the role of genetics while emphasizing the role of environment. Although genetic factors made significant contributions, the environment was the primary contributor to having English (L2) basic literacy difficulties. Shared environmental factors explained 56% of the total variance in probability, of which SES and school language use took up 5% and 15%, respectively. Lower SES and less English language use at school were associated with a higher probability of EPCDL. These findings call for future studies to investigate the mechanisms by which the environment contributes to EPCDL or to specific second language reading and spelling difficulties. Efforts can be made to identify the environmental factors that influence cognitive and neural deviations in children with literacy difficulties. Identifying the environmental factors could set the stage for the subsequent design of interventions.

Although less dominant, genetic factors still made a significant contribution to the probability of EPCDL (see Table 4). In fact, the heritability of EPCDL was not significantly lower than that of PBL. Therefore, we cannot rule out the possibility of common genetic influences on word-level reading/spelling difficulties across language status and scripts. A significant genetic contribution has also been found in a previous study using English and Chinese word reading skills as continuous variables (Wong et al., 2014). These results consistently showed specific genetic contributions to L2 English skills only in Chinese-speaking children. This attests to the validity of specific L2 word reading/spelling difficulties (Miller-Guron & Lundberg, 2000; Tong et al., 2015; Zhang & Wang, 2023), or at the very least so in a bilingual context where L1 and L2 are of great linguistic contrast. Moreover, the genetic contribution to EPCDL was moderated by SES in the same way as PBL (lower SES was associated with greater genetic contribution). Chinese children with a genetic predisposition of having difficulty acquiring English basic literacy may be more likely to become EPCDL in a socioeconomically disadvantaged context.

Other than the difficulty profiles, the present study is among the first to show genetic-versus-environmental aetiologies for language dominance in typically achieving children in a biliterate society. Despite the comparable additive genetic contributions, the CDL and EDL profiles differed significantly in the shared and non-shared environmental contributions. CDL learners had a greater non-shared environmental contribution than EDL learners, whereas EDL learners had a greater shared environmental contribution than CDL learners. This finding points to the factors at the family and school levels that may play a salient role in promoting basic literacy skills in L2 English among Hong Kong Chinese children. This is also confirmed by our results showing that higher SES and more school English use were associated with a higher probability of EDL. Similarly, the literature on academic achievement has shown that environmental factors such as family’s SES, parents’ expectations, and access to additional educational resources exert a stronger influence on L2 English reading than on other outcomes, such as L1 Chinese reading and mathematics skills (e.g., Davis-Kean et al., 2021; Inoue et al., 2023).

### Gene-environment (G × E) interactions

Furthermore, there was a significant interaction between SES and genetic contribution; higher SES was associated with a greater genetic contribution to the probability of EDL. This finding appears to support the Scarr–Rowe effect (Rowe et al., 1999; Scarr, 1992), which posits that higher SES and greater exposure to social advantage during childhood lead to an increase in the heritability of cognitive and academic skills, as compared to children raised in less advantaged environments. However, it is important to note that there have been debates about the generalizability of this hypothesis across contexts. In fact, a meta-analysis has shown that the Scarr–Rowe effect is mostly found in the United States but reduces in size or shows the reversed effect in societies where resources are more equally distributed and good-quality education is widely accessible (Tucker-Drob & Bates, 2016). Our finding of the significant interaction on the probability of EDL, a higher-achieving profile, provides support to the hypothesis in Hong Kong, a city with low economic equality (Gini index of 51.8 in reference to 59.4 in the US and 77.6 in Finland; World Bank, 2022) and yet a good public education system (Gopinathan & Lee, 2018).

Of note, the current study also showed that higher SES was associated with a decreased genetic contribution to probabilities of the difficulty profiles, supporting the vulnerability-stress hypothesis as previously discussed. Although in the opposite direction to the Scarr–Rowe hypothesis, both findings suggest that SES plays a role in moderating the impact of genes on basic biliteracy skills. The finding on EDL highlights how higher SES can amplify genetic potential, whereas the findings on PBL and EPCDL show how lower SES can trigger genetic vulnerabilities (see Catts & Petscher, 2021). Together, they offer a broader framework for understanding individual differences in Chinese-English basic biliteracy development and difficulties in Hong Kong Chinese children.

### Educational implications

The results of the study have some important educational implications. First, the different basic biliteracy difficulty subtypes in the population suggest the need for tailored interventions. Early screening and targeted evidence-based literacy interventions should be implemented for all who need them, and this might be particularly important to mitigate the impact of the genetic risk of poor biliterate learners. The intervention practices may include structured phonics and morphological instruction that support both Chinese and English literacy development (e.g., Colenbrander et al., 2024; Ruan et al., 2024). In turn, English language exposure, particularly school language use, a common environmental factor, may play an important role in English reading development. More educational resources and opportunities should be made widely available to improve English instruction (e.g., immersive English programs) in public schools in biliterate societies like Hong Kong.

Second, lower SES is likely to be a risk factor for basic biliteracy difficulties in Hong Kong children. Lower SES may be associated not only with higher probabilities of both basic biliteracy difficulties but also with an exacerbation of the genetic liability underlying the difficulties. Children with a genetic predisposition to risk for basic literacy difficulties, especially those born in lower SES environments, are more likely to develop difficulties in learning to read and write in their first (Chinese) and second (English) languages. This calls for prioritizing early identification and intervention for at-risk children, such as those from lower SES households and with a family member (sibling or parent) who has basic biliteracy difficulties. To identify these children, schools can consider the family history of English and Chinese basic literacy difficulties, which is not as commonly considered as other demographic information (e.g., SES).

### Limitations and conclusions

Several limitations should be considered when interpreting the findings. First, it should be noted that our classification accuracy of EPCDL learners was relatively lower. Although the mixture of the four distributions best described the heterogeneity in the sample, in practical settings where the priority is risk identification and resource allocation, the label of EPCDL should be used with caution, especially to avoid confusion with CDL learners. Similarly, children with only Chinese literacy difficulties, who did not form as a distinct class in this study, might be mistakenly identified as proficient English learners. Additional measures of Chinese and English basic literacy and related cognitive-linguistic skills may help with the assessment and clinical decisions. Second, we did not consider the gender effect in modelling the genetic versus environmental contributions due to the constraint of sample size. Although the probability of each membership did not differ significantly by gender (χ^2^ = 4.25, *df* = 3, *p* = .236), a same-sex sample would yield a more accurate estimation of aetiology. Third, gene-environment correlations were not fully considered in our analysis, which could have potentially introduced some bias into the results. Further studies with twin-family samples of larger sample sizes are needed, particularly those collecting genetic data simultaneously. Such comprehensive samples would enable advanced analyses, such as genome-wide complex trait analysis (Yang et al., 2011), which can elucidate genetic influences on family- and school-level environments, moving toward an active model of gene-environment interplay.

Despite these limitations, the current study identified previously suggested basic biliteracy difficulty subtypes in a biliterate sample of Hong Kong Chinese twin children and further provided aetiological evidence for different subtypes. Genetic factors explained the majority of the variance in the probabilities of poor biliteracy learners, while environmental factors did so for the probabilities of English-poor only learners. Despite the aetiological heterogeneity, SES appears to be an important risk factor for both difficulty subtypes, increasing the difficulty probabilities and exacerbating genetic liability.

## Supplementary Information

Below is the link to the electronic supplementary material.

Supplementary file1 (DOCX 44 KB)

## Declarations

### Data availability statement

The analysis code is available via this link. https://ln.run/jIbLR The dataset will be provided upon reasonable request.

## References

[CR1] Asparouhov, T., & Muthén, B. (2014). Auxiliary variables in mixture modeling: Three-step approaches using Mplus. *Structural Equation Modeling: A Multidisciplinary Journal,**21*(3), 329–341. 10.1080/10705511.2014.915181

[CR2] Bialystok, E., McBride-Chang, C., & Luk, G. (2005). Bilingualism, language proficiency, and learning to read in two writing systems. *Journal of Educational Psychology,**97*(4), 580–590. 10.1037/0022-0663.97.4.580

[CR3] Boker, S., Neale, M., Maes, H., et al. (2011). OpenMx: An Open Source Extended Structural Equation Modeling Framework. *Psychometrika,**76*, 306–317. 10.1007/s11336-010-9200-6 PMC3525063 23258944

[CR4] Cao, F., Yan, X., Yan, X., Zhou, H., & Booth, J. R. (2021). Reading disability in Chinese children learning English as an L2. *Child Development,**92*(2), e126–e142. 10.1111/cdev.13452 32864778

[CR5] Catts, H. W., & Petscher, Y. (2021). A cumulative risk and resilience model of dyslexia. *Journal of Learning Disabilities*(3). 10.1177/00222194211037062 34365842

[CR6] Catts, H. W., Terry, N. P., Lonigan, C. J., Compton, D. L., Wagner, R. K., Steacy, L. M., Farquharson, K., & Petscher, Y. (2024). Revisiting the definition of dyslexia. *Annals of Dyslexia,**74*(3), 282–302. 10.1007/s11881-023-00295-3 PMC12063701 38194056

[CR7] Cheah, Z. R. E., Ye, Y., Lui, K. F. H., McBride, C., & Maurer, U. (2023). Spelling as a way to classify poor Chinese-English literacy skills in Hong Kong Chinese children. *Annals of Dyslexia,**73*(1), 90–108. 10.1007/s11881-022-00262-4 35763132

[CR8] Colenbrander, D., von Hagen, A., Kohnen, S., Wegener, S., Ko, K., Beyersmann, E., Behzadnia, A., Parrila, R., & Castles, A. (2024). The effects of morphological instruction on literacy outcomes for children in English-speaking countries: A systematic review and meta-analysis. *Educational Psychology Review,**36*(4), 119. 10.1007/s10648-024-09953-3

[CR9] Davis-Kean, P. E., Tighe, L. A., & Waters, N. E. (2021). The Role of Parent Educational Attainment in Parenting and Children’s Development. *Current Directions in Psychological Science,**30*(2), 186–192. 10.1177/0963721421993116

[CR10] Dunn, L. M., & Dunn, L. M (1997). Peabody Picture Vocabulary Test (3rd ed.). Circles Pines, M.N: American Guidance Services.

[CR11] Erbeli, F., Hart, S. A., Wagner, R. K., & Taylor, J. (2018). Examining the etiology of reading disability as conceptualized by the hybrid model. *Scientific Studies of Reading,**22*(2), 167–180. 10.1080/10888438.2017.1407321 PMC5839648 29527114

[CR12] Fletcher, J. M. (1985). External validation of learning disability typologies. In B. P. Rourke (Ed.), *Neuropsychology of learning disabilities: Essentials of subtype analysis* (pp. 187–211). Guilford Press.

[CR13] Fisher, S. E., & DeFries, J. C. (2002). Developmental dyslexia: genetic dissection of a complex cognitive trait. Nature reviews. *Neuroscience, 3*(10), 767–780. 10.1038/nrn936 12360321

[CR14] Gopinathan, S., & Lee, M. H. (2018). Excellence and equity in high-performing education systems: Policy lessons from Singapore and Hong Kong. *Infancia y Aprendizaje,**41*(2), 203–247. 10.1080/02103702.2018.1434043

[CR15] Grigorenko, E. L. (2004). Genetic bases of developmental dyslexia: A capsule review of heritability estimates. *Enfance,**56*(3), 273–288. 10.3917/enf.563.0273

[CR16] Hart, S. A., Logan, J. A. R., Thompson, L., Kovas, Y., McLoughlin, G., & Petrill, S. A. (2016). A latent profile analysis of math achievement, numerosity, and math anxiety in twins. *Journal of Educational Psychology,**108*(2), 181–193. 10.1037/edu0000045 PMC4779361 26957650

[CR17] Hemelstrand, S., & Inoue, T. (2025). Stop splitting hairs: The problems with dichotomizing continuous data in language research. *Research Methods in Applied Linguistics*. 10.1016/j.rmal.2025.100272

[CR18] Ho, C. S. H., Chan, D., Tsang, S. M., Lee, S. H., & Cheng, R. W. Y. (2007). The Hong Kong Test of specific learning difficulties in reading and writing for primary schoolstudents—Second edition (HKT-P[II]). Hong Kong: Hong Kong Specific LearningDifficulties Research Team.

[CR19] Hoff, E., Tulloch, M. K., & Core, C. (2021). Profiles of minority-majority language proficiency in 5-year-olds. *Child Development,**92*(5), 1801–1816. 10.1111/cdev.13591 PMC10399634 34042172

[CR20] Hu, C.-F., & Catts, H. W. (1998). The role of phonological processing in early reading ability: What we can learn from Chinese. *Scientific Studies of Reading,**2*(1), 55–79. 10.1207/s1532799xssr0201_3

[CR21] Huo, S., Wu, K. C., Mo, J., Wang, J., & Maurer, U. (2022). Children with Chinese dyslexia acquiring English literacy: Interaction between cognitive subtypes of dyslexia and orthographies. *Journal of Learning Disabilities,**55*(3), 229–241. 10.1177/00222194211017819 34105413

[CR22] Inoue, T., Zheng, M., Ho, C. S.-H., & McBride, C. (2023). Predicting the developmental trajectories of Chinese reading, English reading, and mathematics: Evidence from Hong Kong Chinese children. *Developmental Psychology, 59*(9), 1652–1667. 10.1037/dev0001575 37410444

[CR23] Jung, T., & Wickrama, K. A. S. (2008). An introduction to latent class growth analysis and growth mixture modeling. *Social and Personality Psychology Compass,**2*(1), 302–317. 10.1111/j.1751-9004.2007.00054.x

[CR24] Li, H., Booth, J. R., Bélanger, N. N., Feng, X., Tian, M., Xie, W., Zhang, M., Gao, Y., Ang, C., Yang, X., Liu, L., Meng, X., & Ding, G. (2018a). Structural correlates of literacy difficulties in the second language: Evidence from Mandarin-speaking children learning English. *NeuroImage,**179*, 288–297. 10.1016/j.neuroimage.2018.06.037 29902587

[CR25] Li, S., Tao, S., Joshi, R. M., & Xu, Q. (2018b). Second-language reading difficulties among native Chinese-speaking students learning to read English: The roles of native- and second-language skills. *Reading Research Quarterly,**53*(4), 423–441. 10.1002/rrq.204

[CR26] Liu, D., Chung, K. K. H., & McBride, C. (2016). The role of SES in Chinese (L1) and English (L2) word reading in Chinese-speaking kindergarteners. *Journal of Research in Reading,**39*(3), 268–291. 10.1111/1467-9817.12046

[CR27] Lo, J. C. M., Ye, Y., Tong, X., McBride, C., Ho, C. S., & Waye, M. M. Y. (2018). Delayed copying is uniquely related to dictation in bilingual Cantonese–English-speaking children in Hong Kong. *Writing Systems Research,**10*(1), 26–42. 10.1080/17586801.2018.1481902

[CR28] Lubke, G. H., & Muthén, B. (2005). Investigating population heterogeneity with factor mixture models. *Psychological Methods,**10*(1), 21–39. 10.1037/1082-989X.10.1.21 15810867

[CR29] Lundberg, I. (2002). Second language learning and reading with the additional load of dyslexia. *Annals of Dyslexia,**52*(1), 165–187. 10.1007/s11881-002-0011-z

[CR30] Mascheretti, S., Bureau, A., Battaglia, M., Simone, D., Quadrelli, E., Croteau, J., Cellino, M. R., Giorda, R., Beri, S., Maziade, M., & Marino, C. (2013). An assessment of gene-by-environment interactions in developmental dyslexia-related phenotypes. *Genes, Brain, and Behavior,**12*(1), 47–55. 10.1111/gbb.12000 23176554

[CR31] McBride-Chang, C., Tardif, T., Cho, J.-R., Shu, H., Fletcher, P., Stokes, S. F., Wong, A., & Leung, K. (2008). What’s in a word? Morphological awareness and vocabulary knowledge in three languages. *Applied Psycholinguistics,**29*(3), 437–462. 10.1017/S014271640808020X

[CR32] McBride-Chang, C., Liu, P. D., Wong, T., Wong, A., & Shu, H. (2012). Specific reading difficulties in Chinese, English, or both: Longitudinal markers of phonological awareness, morphological awareness, and RAN in Hong Kong Chinese children. *Journal of Learning Disabilities,**45*(6), 503–514. 10.1177/0022219411400748 21421936

[CR33] Miller-Guron, L., & Lundberg, I. (2000). Dyslexia and second language reading: A second bite at the apple? *Reading and Writing,**12*(1), 41–61. 10.1023/A:1008009703641

[CR34] Muthén, L. K., & Muthén, B. O. (2017). *Mplus user’s guide* (8th ed). Muthén & Muthén.

[CR35] Neuman, R. J., Heath, A., Reich, W., Bucholz, K. K., Madden, P. A. F., Sun, L., Todd, R. D., & Hudziak, J. J. (2001). Latent class analysis of ADHD and comorbid symptoms in a population sample of adolescent female twins. *Journal of Child Psychology and Psychiatry,**42*(7), 933–942. 10.1111/1469-7610.00789 11693588

[CR36] Nylund, K. L., Asparouhov, T., & Muthén, B. O. (2007). Deciding on the number of classes in latent class analysis and growth mixture modeling: A monte carlo simulation study. *Structural Equation Modeling: A Multidisciplinary Journal,**14*(4), 535–569. 10.1080/10705510701575396

[CR37] Oren, R., & Breznitz, Z. (2005). Reading processes in L1 and L2 among dyslexic as compared to regular bilingual readers: Behavioral and electrophysiological evidence. *Journal of Neurolinguistics,**18*(2), 127–151. 10.1016/j.jneuroling.2004.11.003

[CR38] Petrill, S. A., Pike, A., Price, T., & Plomin, R. (2004). Chaos in the home and socioeconomic status are associated with cognitive development in early childhood: Environmental mediators identified in a genetic design. *Intelligence,**32*(5), 445–460. 10.1016/j.intell.2004.06.010

[CR39] Peugh, J., & Fan, X. (2013). Modeling unobserved heterogeneity using latent profile analysis: A Monte Carlo simulation. *Structural Equation Modeling: A Multidisciplinary Journal, 20*(4), 616–639. 10.1080/10705511.2013.824780

[CR40] Plomin, R. (1990). *Nature and nurture: An introduction to human behavioral genetics*. Thomson Brooks/Cole Publishing Co.

[CR41] Purcell, S. (2002). Variance components models for gene-environment interaction in twin analysis. *Twin Research: The Official Journal of the International Society for Twin Studies,**5*(6), 554–571. 10.1375/136905202762342026 12573187

[CR42] R Core Team (2023) R: A language and environment for statistical computing.* R Foundation for Statistical Computing*, Vienna. https://www.R-project.org/

[CR43] Ramaswamy, V., Desarbo, W. S., Reibstein, D. J., & Robinson, W. T. (1993). An empirical pooling approach for estimating marketing mix elasticities with PIMS data.* Marketing Science, 12*(1), 103–124. http://www.jstor.org/stable/183740

[CR44] Rende, R., & Plomin, R. (1992). Diathesis-stress models of psychopathology: A quantitative genetic perspective. *Applied and Preventive Psychology,**1*(4), 177–182. 10.1016/S0962-1849(05)80123-4

[CR45] Rowe, D. C., Jacobson, K. C., & Van Den Oord, E. J. C. G. (1999). Genetic and environmental influences on vocabulary IQ: Parental education level as moderator. *Child Development,**70*(5), 1151–1162. 10.1111/1467-8624.00084 10546338

[CR46] Ruan, Y., Maurer, U., & McBride, C. (2024). Effectiveness of reading interventions on literacy skills for Chinese children with and without dyslexia: A meta-analysis of randomized controlled trials. *Educational Psychology Review,**36*(3), 1–40. 10.1007/s10648-024-09915-9

[CR47] Scarr, S. (1992). Developmental theories for the 1990s: Development and individual differences. *Child Development,**63*(1), 1–19. 10.1111/j.1467-8624.1992.tb03591.x 1343618

[CR48] Sparks, R. L. (1995). Examining the linguistic coding differences hypothesis to explain individual differences in foreign language learning. *Annals of Dyslexia,**45*(1), 187–214. 10.1007/BF02648218 24234194

[CR49] Tong, X., McBride-Chang, C., Shu, H., & Wong, A.M.-Y. (2009). Morphological awareness, orthographic knowledge, and spelling errors: Keys to understanding early Chinese literacy acquisition. *Scientific Studies of Reading,**13*(5), 426–452. 10.1080/10888430903162910

[CR50] Tong, X., & McBride-Chang, C. (2010). Developmental models of learning to read Chinese words. *Developmental Psychology,**46*(6), 1662. 10.1037/a0020611 20836598

[CR51] Tong, X., Tong, X., & McBride-Chang, C. (2015). A tale of two writing systems: Double dissociation and metalinguistic transfer between Chinese and English word reading among Hong Kong children. *Journal of Learning Disabilities,**48*(2), 130–145. 10.1177/0022219413492854 23784785

[CR52] Tucker-Drob, E. M., & Bates, T. C. (2016). Large cross-national differences in gene × socioeconomic status interaction on intelligence. *Psychological Science,**27*(2), 138–149. 10.1177/0956797615612727 PMC4749462 26671911

[CR53] Van Buuren, S. (2018) *Flexible Imputation of Missing Data*. 2nd Edition*,* CRC Press, Boca Raton. 10.1201/9780429492259

[CR54] Van Der Leij, A., & Morfidi, E. (2006). Core deficits and variable differences in Dutch poor readers learning English. *Journal of Learning Disabilities,**39*(1), 74–90. 10.1177/00222194060390010701 16512084

[CR55] Wong, S. W. L., Chow, B. W. Y., Ho, C. S. H., Waye, M. M. Y., & Bishop, D. V. M. (2014). Genetic and environmental overlap between Chinese and English reading-related skills in Chinese children. *Developmental Psychology,**50*(11), 2539–2548. 10.1037/a0037836 PMC4221000 25221842

[CR56] World Bank. (2022). World development indicators. World Bank. https://databank.worldbank.org/source/world-development

[CR57] Xie, Q., Zheng, M., Ho, C. S. H., McBride, C., Fong, F. L. W., Wong, S. W. L., & Chow, B. W. Y. (2022). Exploring the genetic and environmental etiologies of phonological awareness, morphological awareness, and vocabulary among Chinese-English bilingual children: The moderating role of second language instruction. *Behavior Genetics,**52*(2), 108–122. 10.1007/s10519-021-10096-2 35020106

[CR58] Yang, J., Lee, S. H., Goddard, M. E., & Visscher, P. M. (2011). GCTA: a tool for genome-wide complex trait analysis.* The American Journal of Human Genetics, 88(*1), 76–82. 10.1016/j.ajhg.2010.11.011 PMC3014363 21167468

[CR59] Zhang, J., & Wang, Q. (2023). Reading and writing difficulties in bilingual learners. *Annals of Dyslexia,**73*(1), 1–5. 10.1007/s11881-023-00282-8 37071318

