# Supplementary material for: Aetiologies of basic biliteracy difficulties in Hong Kong Chinese children: A latent profile analysis with twin data
Source: Ann Dyslexia. 2025 Dec 5;76(3):460–81. doi: 10.1007/s11881-025-00355-w (PMC13759662; doi:10.1007/s11881-025-00355-w)
Supplement: Supplementary file 1 [file 11881_2025_355_MOESM1_ESM.docx]

Table S1 The school and home language use of the current sample

|  | Levels | % | n | mean | sd |
| --- | --- | --- | --- | --- | --- |
| Grade | G1 | 53.0 | 479 |  |  |
|  | G2 | 21.0 | 190 |  |  |
|  | G3 | 14.3 | 129 |  |  |
|  | G4 | 11.7 | 106 |  |  |
| Family Language* | Cantonese | 82.0/81.6/82.3/46.7 | 741/738/734/422 |  |  |
|  | Mandarin | 5.5/6.6/5.3/24.9 | 50/60/48/225 |  |  |
|  | English | 1.8/0.7/3.5/19.5 | 16/6/32/175 |  |  |
|  | Chinese Dialect | 1.1/0.7/0.7/2.7 | 10/6/6/27 |  |  |
|  | Other | 1.8/2.4/1.4 | 16/22/14/0 |  |  |
|  | Cantonese&Mandarin | 0.9/1.4/0.7 | 8/13/6/0 |  |  |
|  | Cantonese&English | 1.7/0.7/1.1 | 15/6/10/0 |  |  |
|  | Cantonese and Chinese Dialect | 0.4/0.2/0.2 | 4/2/3/0 |  |  |
| Primary school main language of instruction | Cantonese | 65.6 | 593 |  |  |
|  | Mandarin | 3.9 | 35 |  |  |
|  | English | 8.7 | 79 |  |  |
| # of subjects^+^ instructed in  Cantonese | | | | 4.14 | 1.29 |
| # of subjects instructed in  Mandarin | | | | 0.59 | 0.76 |
| # of subjects instructed in  English | | | | 0.57 | 1.14 |
| English tutor | yes | 45.9 | 415 |  |  |
|  | no | 48.0 | 434 |  |  |
| English Phonics | yes | 32.7 | 296 |  |  |
|  | no | 59.4 | 537 |  |  |

Note. * father language/mother language/sibling language/helper language;+ , the subjects other than the language subject. All children in the sample received language lessons in Chinese (Cantonese/Mandarin) and English at school

we examined whether the latent class membership could cluster in families, and further tease apart genetic versus environmental influences as follows. First, we calculated the affected-status agreement statistic of individuals in a given class being concordant in that class with their co-twin and the confidence intervals. The affected-status agreement statistic larger than 50% indicates being in that class is driven by a combined influence of shared familial environment and genes (Hart et al., 2015). We then computed the odds ratio of being assigned to the same latent class conditioned on zygosity, MZ versus DZ, and its 95% confidence interval. The odds ratio significantly larger than 1 indicates significant genetic influence (Neuman et al., 2001).

**Table S2 Family Agreement Status of Each Profile and Confidence Interval**

| Profile | Family agreement status | SE | Twin concordance odds ratio |  |
| --- | --- | --- | --- | --- |
| Poor biliterate | 69.6% [.62; .77] | 0.04 | 2.400 [1.054;5.458] |  |
| Chinese dominant | 63.7% [.59; .69] | 0.03 | 1.399 [.854;.2.293] |  |
| English dominant | 75.8% [.70; .82] | 0.03 | .737 [.344;1.581] |  |
| Poor English learners | 54.8% [.47; .62] | 0.04 | 1.395 [.669;2.910] |  |

**Table S3 Fitness indices of fully variant models, partially-variant and non-variant models**

| Model | Classes | LogLik | AIC | BIC | SABIC | BLRT_val | BLRT_p |
| --- | --- | --- | --- | --- | --- | --- | --- |
| Fully variant | 1 | -4094.65 | 8217.30 | 8284.59 | 8240.13 | - | - |
| Fully variant | 2 | -3888.20 | 7834.40 | 7973.79 | 7881.69 | 412.90 | 0.00 |
| Fully variant | 3 | -3809.05 | 7706.10 | 7917.60 | 7777.87 | 158.29 | 0.00 |
| Fully variant | 4 | -3767.92 | 7653.84 | 7937.44 | 7750.07 | 82.26 | 0.00 |
| Fully variant | 5 | -3751.94 | 7651.88 | 8007.58 | 7772.57 | 44.03 | 0.43 |
| Fully variant | 6 | -3723.93 | 7625.86 | 8053.67 | 7771.02 | 82.39 | 0.00 |
| Partially variant 1 | 1 | -4094.65 | 8217.30 | 8284.59 | 8240.13 | - | - |
| Partially variant 1 | 2 | -3961.71 | 7969.42 | 8079.98 | 8006.93 | 265.88 | 0.00 |
| Partially variant 1 | 3 | -3845.36 | 7754.73 | 7908.54 | 7806.92 | 232.69 | 0.00 |
| Partially variant 1 | 4 | -3807.63 | 7697.26 | 7894.34 | 7764.13 | 75.47 | 0.00 |
| Partially variant 1 | 5 | -3774.70 | 7649.40 | 7889.74 | 7730.95 | 65.86 | 0.00 |
| Partially variant 1 | 6 | -3763.11 | 7644.22 | 7927.83 | 7740.45 | 23.18 | 1.00 |
| Partially variant 2 | 1 | -4094.65 | 8217.30 | 8284.59 | 8240.13 | - | - |
| Partially variant 2 | 2 | -3974.55 | 7999.11 | 8119.28 | 8039.88 | 240.19 | 0.00 |
| Partially variant 2 | 3 | -3865.93 | 7803.87 | 7976.91 | 7862.58 | 217.24 | 0.00 |
| Partially variant 2 | 4 | -3818.29 | 7730.58 | 7956.50 | 7807.24 | 95.28 | 0.00 |
| Partially variant 2 | 5 | -3787.08 | 7690.16 | 7968.96 | 7784.76 | 59.87 | 0.00 |
| Partially variant 2 | 6 | -3766.51 | 7671.02 | 8002.69 | 7783.56 | 78.94 | 0.00 |
| Invariant | 1 | -4094.65 | 8217.30 | 8284.59 | 8240.13 | - | - |
| Invariant | 2 | -3993.76 | 8025.52 | 8116.85 | 8056.51 | 201.78 | 0.00 |
| Invariant | 3 | -3926.14 | 7900.28 | 8015.65 | 7939.43 | 135.24 | 0.00 |
| Invariant | 4 | -3896.03 | 7850.05 | 7989.45 | 7897.35 | 60.23 | 0.00 |
| Invariant | 5 | -3873.96 | 7815.91 | 7979.35 | 7871.37 | 44.14 | 0.00 |
| Invariant | 6 | -3868.41 | 7814.82 | 8002.29 | 7878.43 | 11.10 | 1.00 |

**Table S4**

**Profile and Demographic Characteristics of 3- to 5-profile Solutions**

|  | Class proportion | EWR | CWR | EDICT | CDICT | Age | Grade |
| --- | --- | --- | --- | --- | --- | --- | --- |
|  | 3-class solution | | | | | | |
| Low biliterate (*n* = 134) | 14.80% | -0.97 | -1.26 | -0.76 | -0.95 | 81.78 | 1.66 |
| Chinese dominant  (*n* = 456) | 50.40% | -0.60 | 0.23 | -0.48 | 0.21 | 92.92 | 2.55 |
| English dominant (*n* = 314) | 34.70% | 1.17 | 0.22 | 0.96 | 0.33 | 92.18 | 2.47 |
|  | 4-class solution | | | | | | |
| Low biliterate (*n* = 154) | 17.00% | -0.94 | -1.19 | -0.73 | -0.91 | 81.88 | 1.66 |
| Chinese dominant (*n* = 463) | 51.20% | -0.03 | 0.32 | -0.12 | 0.35 | 91.95 | 2.47 |
| English dominant (*n* = 175) | 19.40% | 1.43 | 0.13 | 1.49 | 0.44 | 94.38 | 2.64 |
| Poor English learners (*n* = 112) | 12.40% | -1.15 | 0.14 | -0.99 | -0.25 | 94.41 | 2.66 |
|  | 5-class solution | | | | | | |
| Chinese dominant (*n* = 445) | 49.23% | -0.61 | 0.15 | -0.54 | 0.17 | 92.64 | 2.52 |
| English dominant (*n* = 140) | 15.49% | 1.06 | 0.38 | 0.47 | 0.04 | 87.99 | 2.11 |
| Balanced (*n* = 71) | 7.85% | 0.51 | 0.72 | 0.89 | 0.79 | 96.72 | 2.91 |
| Low biliterate (*n* = 127) | 14.05% | -0.97 | -1.29 | -0.76 | -0.98 | 81.66 | 1.66 |
| Highly English dominant (*n* = 121) | 13.38% | 1.43 | -0.04 | 1.55 | 0.50 | 94.95 | 2.71 |

**Table S5-1	Transitional Matrix from 3-class to 4-class**

| 4-class solution  3-class solution | 1. Low biliterate | 2. Chinese dominant | 3. English dominant | 4. Poor English learners |
| --- | --- | --- | --- | --- |
| 1. Low biliterate | 133 | 0 | 0 | 1 |
| 2. Chinese dominant | 21 | 324 | 0 | 111 |
| 3.English dominant | 0 | 139 | 175 | 0 |

**Table S5-2	Transitional Matrix from 4-class to 5-class**

| 5-class solution  4-class solution | 1. Low biliterate | 2. English dominant | 3. Balanced | 4. Chinese dominant | 5. English dominant good learners |
| --- | --- | --- | --- | --- | --- |
| 1. Low biliterate | 126 | 0 | 0 | 28 | 0 |
| 2. Chinese dominant | 0 | 111 | 44 | 306 | 2 |
| 3. English dominant | 0 | 29 | 27 | 0 | 119 |
| 4. Poor English learners | 1 | 0 | 0 | 111 | 0 |

**Table S5-3	Transitional Matrix from 3-class to 5-class**

| 5-class solution  3-class solution | 1. Low biliterate | 2. English dominant | 3. Balanced | 4. Chinese dominant | 5. English dominant good learners |
| --- | --- | --- | --- | --- | --- |
| 1. Low biliterate | 127 | 0 | 0 | 7 | 0 |
| 2. Chinese dominant | 0 | 4 | 30 | 422 | 0 |
| 3. English dominant | 0 | 136 | 41 | 16 | 121 |

**Table S6**

**Descriptive statistics on the final solution of the classification analysis and comparison on classification, external, and demographic variables**

|  | PBL | CDL | EDL | EPL | *F* | post hoc test |
| --- | --- | --- | --- | --- | --- | --- |
| n(n_boys_), % | 161(73), 17.80% | 377 (201),  41.7% | 198 (92), 21.9% | 168 (87),  18.6% | |  |
|  | Classification variables | | |  |  |  |
| zEWR | -0.93(0.22) | 0.03(0.72) | 1.36(0.46) | -0.99(0.48) | 11.94*** | PBL,EPL<CDL<EDL |
| zCWR | -1.17(0.57) | 0.26(0.91) | 0.18(1.00) | 0.35(0.86) | 143.11*** | PBL<CDL,EPL,EDL |
| zEDICT | -0.72(0.24) | -0.10(0.59) | 1.42(0.45) | -0.87(0.43) | 15.854*** | PBL<CDL,EPL<EDL |
| zCDICT | -0.88(0.81) | 0.30(0.87) | 0.43(0.85) | 0.08(0.99) | 62.74*** | PBL<EPL<CDL,EDL |
|  | External variables | |  |  |  |  |
| zEVK | -0.74(0.58) | -0.02(0.90) | 1.07(0.22) | -1.00(0.91) | 321.21*** | EPL<PBL<CDL<EDL |
| zCOM | -0.85(0.22) | 0.14(0.22) | 0.23(0.22) | 0.13(0.22) | 64.25*** | PBL<CDL,EPL,EDL |
|  | Background information | | |  |  |  |
| EMOI | -0.29(0.59) | -0.18(0.75) | 0.79(1.23) | -0.34(0.35) | 6.28* | PBL, CDL, EPL>EDL |
| CMOI | 0.20(0.61) | 0.17(0.75) | -0.67(1.23) | 0.32(0.37) | 12.43*** | PBL, CDL, EPL < EDL |
| HomeLan | -0.89(0.23) | -0.85(0.26) | -0.77(0.33) | -0.89(0.22) | 0.6 |  |
|  |  |  |  |  |  |  |
| Eng tutor | -0.16(0.96) | 0.11(1.02) | 0.21(1.00) | -0.22(0.90) | 0.12 |  |
| Fedu | 3.44(1.86) | 4.01(1.75) | 5.08(1.40) | 3.06(1.57) | 0.05 |  |
| Medu | 3.44(1.46) | 3.92(1.66) | 4.92(1.60) | 2.92(1.50) | 1.28 |  |
| Income | 3.85(1.67) | 4.59(1.68) | 5.58(0.94) | 3.39(1.60) | 0.01 |  |

Note. PBL, Poor biliterate learner; CDL, Chinese dominant learners; EDL, English dominant learners; EPL, English poor learners; EMOI, the amount of school instruction mediated in English standardized in z score; CMOI, the amount of school instruction mediated in Chinese standardized in z score; CWR, Chinese word reading; EWR, English word reading; CDICT, Chinese dictation; EDICT, English dictation; COM, Chinese one minute word reading; EVOC, English vocabulary knowledge; HomeLan, home language, -1 indicates completely Chinese, 1 indicates completely English; Eng tutor, the amount of English tutoring; Fedu, father educational obtainment, 7, Doctoral Degree, 6, Master's Degree , 5, Bachelor's Degree, 4, Post Secondary (Associate Degree or Higher Diploma), 3, Form 5 to Form 7, 2 Form 3 to Form 5, 1, Form 3 or below; Medu, father educational obtainment; Income, 6, $50,001 or above ; 5, $40,001-$50,000 ;4, $30,001-$40,000; 3 , $20,001-$30,000; 2; $10,001-$20,000; 1, Less than $10,000.

Table S7-1 Tabulated table between the difficulty class membership yielded from the cut-off approach on word reading and the membership yielded from our final LPA model

|  | Poor biliterate | Chinese Dominant | English Dominant | English Poor Only |
| --- | --- | --- | --- | --- |
| Typical | 48 | 301 | 157 | 79 |
| Difficulty in English | 1 | 33 | 0 | 79 |
| Difficulty in Chinese | 0 | 24 | 41 | 0 |
| Difficulty in both | 112 | 19 | 0 | 22 |

Table S7-2 Tabulated table between the difficulty class membership yielded from the cut-off approach on word dictation and the membership yielded from our final LPA model

|  | Poor biliterate | Chinese Dominant | English Dominant | English Poor Only |
| --- | --- | --- | --- | --- |
| Typical | 86 | 302 | 174 | 76 |
| Difficulty in English | 21 | 38 | 0 | 61 |
| Difficulty in Chinese | 0 | 23 | 24 | 0 |
| Difficulty in both | 54 | 14 | 0 | 35 |
